# Understanding Intimate Partner Violence: Why Coercive Control Requires a Social and Systemic Entrapment Framework

**DOI:** 10.1177/10778012231205585

**Published:** 2023-10-09

**Authors:** Julia Tolmie, Rachel Smith, Denise Wilson

**Affiliations:** 1 1415 University of Auckland, Auckland, New Zealand; 2 1410 Auckland University of Technology, Auckland, New Zealand

**Keywords:** intimate partner violence, coercive control, entrapment

## Abstract

How intimate partner violence (IPV) is conceptualized affects what we see when we look at situations involving IPV and what we think the solutions to the problem of IPV are—either in individual cases or in the development of broader legal and policy responses. In this article, it is suggested that while conceptualizing IPV as coercive control is an improvement over previous understandings, it does not go far enough. Coercive control must be located within a broader conceptualization of IPV as a form of social and systemic entrapment if it is not to operate in a harmful manner for victim-survivors.

## Introduction

How one conceptualizes intimate partner violence (IPV) influences what is seen when looking at situations involving IPV. It affects what is considered relevant or irrelevant in making sense of what is happening and the meanings derived from factors deemed “relevant.” It also influences what one thinks is the appropriate response to IPV—either in a specific instance or in the development of laws and policies to respond to IPV more generally. In this article, we explain why conceptualizing IPV as coercive control does not go far enough, despite being an improvement on previous frameworks. We suggest that locating coercive control within a broader understanding of social and systemic entrapment is critical in legal, policy, and practical contexts to avoid potentially harmful conceptualizations of IPV. This article deepens the understanding of IPV entrapment (see Tolmie et al., 2018) by highlighting the significance of socio-cultural and structural issues in the hierarchy of analysis.

It is important to note that by entrapment, we do not mean entrapment *in the relationship* with an abusive partner. As explained later, we include in the concept of IPV entrapment the effects of state neglect and violence toward victim-survivors of IPV and their families and communities. By entrapment, we are referring to the restrictions placed on a victim-survivor's autonomy and agency by their *partner's abusive and controlling behaviors* and by *broader systemic patterns of harm*, which frequently continue even after a victim-survivor leaves their abusive partner (Toews & Bermea, 2017).

Entrapment broadens the understanding of IPV as coercive control in several ways. It requires a realistic consideration of the safety responses available to the victim-survivor. Entrapment also requires an understanding of how the intersection of inequities associated with race, class, sex, gender, sexual preference, and other forms of oppression in the victim-survivor's life (within the overall matrix of colonization for indigenous victim-survivors in settler states) mediate their experience of both their partner's coercive control and the safety responses available to them. Essentially, entrapment focuses not solely on their partner's abusive behaviors but also on how these play out in the broader social context occupied by the victim-survivor and their abusive partner. Far from operating in a one-size-fits-all manner, entrapment demands careful attention to be paid to the specificity of the victim-survivor's current social location, as well as their and their family's historical and intergenerational life experiences.

Although IPV entrapment is a concept that is useful for *all* victims of IPV, there are particularly harmful consequences in responding to IPV solely as an issue of coercive control for Indigenous peoples and marginalized groups targeted for oppression. A narrative of IPV that focuses on individuals renders invisible the state violence, institutional abuse, and the violence of social exclusion that many victim-survivors, their families, and communities experience (Richie et al., 2021). These latter forms of violence drive the overrepresentation of people subjected to intersecting forms of oppression in family violence statistics and greatly impede their safety, healing, and ability to be self-determining (Brown et al., 2022).

This article is divided into three parts. First, we briefly examine traditional Western approaches to understanding IPV that have dominated the legal context and, increasingly, specialist domestic abuse practitioners’ practice settings. These use battered woman syndrome and its neoliberal reincarnation, trauma-informed practice, to understand victim-survivors’ experiences and their responses to IPV. We explain how these approaches serve to erase the violence and the inadequacy of social responses to this form of violence. These factors ultimately recast victim-survivor's perceptions of, and their responses to, IPV as irrational.

In the second part, we acknowledge that the concept of coercive control represents a significant improvement over traditional Western mental health approaches to understanding IPV. This is because it shifts the focus for understanding the victim-survivor's experiences of, and reactions to, IPV from their mental and physiological processes onto their abusive partner's behavior. It also allows a more complete picture of the range and operation of the abuse tactics over time, explaining how these tactics cumulatively and over time close down the victim-survivor's “space for action” (Sharp-Jeffs et al., 2018) and severely restrict their autonomy. It is important to note that we do not understand coercive control as being synonymous with psychological abuse. Instead, we adopt Evan Stark's position that physical violence or the threat of physical violence is a tactic of coercive control, which is frequently, although not always, present (Barlow & Walklate, 2022; Stark, 2007).

In the third part of this article, we explain why locating coercive control within a broader understanding of social and systemic entrapment is useful for all victim-survivors, but particularly for victim-survivors living at the extreme intersection of intimate partner and structural violence. These victim-survivors require equitable and ultimately transformative safety and justice responses (Kim, 2018).

At the end of each part of this article, we illustrate the approach taken by referring to the experiences of Terri Friesen, a wāhine Māori (indigenous woman in Aotearoa New Zealand) who has given us permission to tell her story. We aim to use her story to demonstrate, in a practical manner, how one's understanding of, and response to, a set of facts involving IPV shifts depending on the manner in which IPV is conceptualized, and therefore, the scope of the facts gathered and the lens used to analyze those facts.

Our language and examples in this article draw from the experiences of cisgender heterosexual women because they are the majority of IPV victim-survivors, particularly in relation to the severity and lethality of abuse (see FVDRC, 2017, p. 9). However, we note that the concept of IPV entrapment is useful in framing an investigation into the experiences of IPV for all victim-survivors, including those who identify as gender-diverse or as cisgender men.

## Battered Woman Syndrome and Trauma-Informed Practice: Pathologizing the Victim

Traditionally, within the criminal justice system (and in other contexts, such as child protection, the family court, and parts of the family violence sector), IPV was understood and responded to as a series of assault crimes, in between which adult victim-survivors were considered free to make safety decisions (Stark, 2013, p. 19). Gore (2022, p. 8) has pointed out that female victim-survivors are often implicitly understood and responded to as “entrepreneurial subjects” who are responsible for failing to make reasonable life choices in order to achieve safety for themselves or their children.

Lawyers seeking to explain why victim-survivors failed to make rational safety decisions and therefore found themselves in unsafe situations (in other words, why they did not leave their abusive partner or get help) have looked to mental health professionals, such as psychologists and psychiatrists, to explain victim-survivor's decision-making processes. Battered woman syndrome (Walker, 1997) represented an early attempt to provide such an explanation. It has been extensively critiqued in the academic literature for understanding a victim-survivor's experiences of IPV in terms of her psyche, rather than emphasizing the violence and the systemic response to that violence (Stubbs & Tolmie, 1995). Despite such critique, evidence on battered woman syndrome is still being used in criminal courts by the psychological and psychiatric professions, well-intentioned defense counsel, and judges (see *Liyanage v. Western Australia*, 2017).

Concerningly, battered woman syndrome is reappearing with the same reductionist pathology under the new guise of trauma-informed practice. Trauma-informed health approaches were originally intended to be responsive to victim-survivors’ suffering (Sweeney et al., 2018) and to explain why current events can trigger past experiences of trauma when victim-survivors have conditions like post-traumatic stress disorder. Such approaches also provide physiological explanations for how people respond to danger—for example, Cannon's “fight or flight” theory (with the latter additions of “freeze” and “fawn”) explains how animals react to threats with a general discharge of the sympathetic nervous system (Cannon, 1929).

The danger of using a trauma lens to understand IPV is that, like the use of battered woman syndrome, it risks erasing the violence the victim-survivor was responding to and makes their trauma the object of inquiry, decontextualized from the lived reality of their social context. In this rendering, it is all too easy to overlook the danger they were in and, instead, understand the victim-survivor's behavior as a consequence of their biology or faulty mental functioning. For example, it can be assumed that a victim-survivor was too traumatized to contact services when actually they do not trust government services because these services have inappropriately responded or have not been responsive to them or their community. Or it can be assumed fear caused them to freeze rather than call the police, when in reality calling the police would have likely increased the level of danger they had to deal with or it was too dangerous to make a phone call. Or their “overreaction” to their abusive partner's use of violence can be explained on the basis that they were triggered by a childhood trauma history, when in fact they knew their partner was trying to kill them and they were acting to defend themselves.

Focusing on the activation of the victim-survivor's nervous system in response to life-threatening situations or triggers related to their trauma history means that we may fail to appreciate their responses as reasonable in the circumstances. In this framing, victim-survivors are not considered active safety strategists attuned to danger through their situational intelligence and experiential knowledge (often derived from navigating years of abuse). Instead, trauma becomes an amorphous one-size-fits-all explanation for their experience of and response to IPV—like any Western psychological approach, typically resulting in a focus on their deficits in thinking and responding.

Of course, this is not to suggest that victim-survivors do not experience emotional, mental, spiritual, cultural, and physiological distress due to their experiences of violence, but rather that their experiences of distress should not be (mis)used in reductionist and deterministic ways to flatten and (re)define their nuanced anticipatory responses to violence (Johnstone et al., 2018). People commit violence, not trauma, and victims-survivors are responding to and resisting violence, not trauma.

Trauma discourse has been criticized by IPV health researchers due to its inadequate recognition of structural and interpersonal violence (Wathen & Mantler, 2022). It has also been criticized by indigenous and critical race scholars for bolstering neoliberal and colonial interventions for fixing faulty individuals, rather than addressing settler state violence, which is why Clark (2016) stated that trauma is the “new colonial frontier” (p.14).

### Case Example: Terri Friesen

In 1989, Terri Friesen was a 21-year-old living with her 2-year-old daughter (Rebecca) and her partner (Brownie Broughton) when she gave birth to their baby, Chantelle. Brownie was violent toward Rebecca and Terri. One morning, they discovered Chantelle dead. Terri assumed it was a cot death. However, a post-mortem revealed that Chantelle died from an intracranial hemorrhage that indicated trauma.

Both parents were taken to the police station and interrogated separately. Terri was informed that Chantelle's death was not a cot death when one of the police officers picked up a child's doll and shook it violently in front of her, saying that this is what happened to her baby. Terri was “numb and stunned, trying to process what was going on…”^1^ She did not have legal counsel and did not realize that she was free to refuse to answer questions and leave. The police were dealing with a weeping father and a mother who was not crying despite having lost a baby. They decided Terri was responsible for Chantelle's death.

The police told Terri that if Brownie went to jail, he might be killed, while the legal system would likely be lenient toward her. They also threatened to remove Rebecca (who was not Brownie's daughter) from her and put her in state care or send her home alone with Brownie if Terri did not confess. Terri did not trust Rebecca in Brownie's care because he was violent toward her. Terri also believed that if Rebecca was taken into state care, she would be molested, possibly physically abused, and could even die. For Terri, this was too much, and she falsely confessed to shaking Chantelle. She was charged with manslaughter and released on bail—meaning that she was able to take Rebecca home and had the opportunity to make plans for Rebecca to be cared for by her mother during court proceedings. At trial, she was convicted of manslaughter and then spent 6 weeks in prison before being sentenced to a 6-month community sentence.

In 1991, Brownie confessed to the police that he had shaken the baby, but they decided not to reopen the case. Eleven years later, Brownie confessed again. In 2002, he pleaded guilty to and was convicted and sentenced for manslaughter. The sentencing judge stated, “Chantelle's mother in fact had nothing to do with Chantelle's death. You caused Chantelle's death” (*R v. Broughton*, 2002, p. 6).

Terri's conviction, however, was not automatically quashed. Nearly 30 years after her original conviction, she managed to assemble a pro bono legal team, and the Court of Appeal in 2018 quashed Terri's conviction (Friesen v. R, 2018). Terri had carried the social stigma of a conviction for all this time: “She was shunned by her neighbors, monitored by the local police, and rejected by potential employers who did not want to hire a woman guilty of manslaughter.”^2^ She was forced to rely on the state for survival. Her tamariki (children) also bore the consequences of her conviction because everyone soon realized who their mother was.

In 2018, Terri sought compensation for her wrongful conviction and imprisonment. She did so on legal advice and to address her extreme poverty for the sake of her tamariki and mokopuna (grandchildren). In the application, her lawyer included a report from a psychologist to the effect that Terri's “particular mental vulnerabilities as a battered spouse” had a “significant influence on her false confession to the police”.^3^ It was noted that “a battered woman might react to questioning by a police officer or other person in authority in a manner which is similar to the way in which she responded to her abuser.”^4^ This is because she may be compliant and provide what she believes is wanted from her.

The Minister of Justice declined Terri's application. Although the 6 weeks she served in prison made her eligible for compensation, the Minister said that she did not meet either of the two steps in the relevant test—that she was innocent on the balance of probabilities and that compensation was in the interests of justice. Of significance to both issues was her own confession which “was both credible and consistent with the evidence and was not contributed to by a diagnosed mental disorder.” The Minister stated that even if she were innocent

Wanting to protect her husband from prison and daughter from state care explain why she falsely confessed but does not excuse it. The integrity of the criminal justice system depends on participants telling the truth. Intentionally lying to Police and the Courts perverts the course of justice.^5^

While the Minister characterized Terri's confession as evidence of her guilt or dishonesty, her lawyer argued that her confession was false and made only because of her fragile mental state as a victim. On both sides of this dispute, the presence or absence of mental health issues due to IPV victimization is key in determining whether Terri experienced a miscarriage of justice.

## Coercive Control: Focusing on the Abuse Tactics

Stark describes IPV as a liberty crime. In other words, it is not about physically hurting the victim-survivor but it is “a course of conduct: a repeated pattern of behavior” (Barlow & Walklate, 2022, pp. 1–2) directed at diminishing the victim-survivor's autonomy and limiting their space for action. This is achieved by the use of tactics of coercive control, developed by trial and error over time by the person who knows the victim-survivor most intimately. It follows that while we can describe the general infrastructure of coercive control, its expression is unique to the people involved and the circumstances of their lives.

Stark (2007), who has been most influential in defining coercive control, details how it employs both indirect and direct tactics. Indirect tactics of control—isolation, deprivation, exploitation, and microregulation—undermine the victim-survivor's independence and foster a dependence on the person using violence. Direct tactics of coercion—violence and intimidation—compel the victim-survivor to appease their abusive partner by forcing compliance and eroding their “will to resist”. Properly employed, coercive control therefore allows us to move beyond understanding IPV as confined to the incidents of physical abuse, between which the victim-survivor is assumed to be free from abuse. It is important to note that victim-survivors always resist the abuse (Kinewesquao (Richardson & Wade, 2009)—although this resistance may be covert when they are not able to bear the costs of overt resistance.

Coercive control is not so much about the particular behaviors used, as it is about their effect in combination and repetition over time. Hamberger et al. (2017) pointed out it is the “chronicity and pervasiveness” of coercive control that is relevant to understand (p. 10). For example, Stark (2007) said that the use of *retaliatory* physical violence can be “low level” repetitive violence that wears the victim-survivor down—she is “bearing the weight of multiple harms” (p. 94).

The concept of coercive control has been hugely influential in shifting thinking about the nature of IPV—finding expression in government policy, legislation, and academic writing around the world. The advantage of a coercive control framing is that it makes a partner's abusive behavior *fully visible*, thus rendering the victim-survivor's perceptions and reactions to their abusive partner's behavior understandable and not informed by psychopathology. Furthermore, we can locate the victim-survivor's perceptions of the abuse and their safety options on any one occasion in the context of the overall and ongoing pattern of harmful behaviors that they are experiencing, have experienced, and will likely experience.

Because the tactics of coercive control are developed by trial and error for the particular victim-survivor, it is not a one-size-fits-all model for understanding IPV. Furthermore, it requires an inquiry that is broader than simply focusing on the mental processes of a particular victim-survivor. This makes the concept of coercive control difficult to translate into legal and policy responses. Certainly, legal actors remain comfortable with traditional understandings of IPV, preferring to frame the issue in terms of the victim-survivor's mental health and distrusting expert testimony in court that does not come from psychologists and psychiatrists (*Liyanage v. Western Australia*, 2017; Sheehy, 2018). The risk is that coercive control will be heard in the legal process simply as another way to understand what has gone into causing the victim's *mental vulnerability* (see *R v. Challen*, 2019).

### Case Example: Terri Friesen

Terri Friesen and her close friends and whānau (extended family networks) have spoken about how Brownie isolated Terri. She was not allowed to socialize or even go shopping. Her mother would park down the end of the road, hoping to catch a glimpse of Terri or the children. Terri would look at the ground when people were around because she was not permitted to look at anyone.

Terri described Brownie as “a control freak” and others referred to his “psychological manipulation.”^6^ Terri kept the house immaculate, was immaculate herself, and tried to school the children to be perfect, “and yet no matter what” Brownie would hit her. Police officers visiting after Chantelle's death observed that the house was extremely clean—it was as though a child did not live there.

Brownie's abuse of Terri was described as “harrowing reading, recounting rape … [and] brutal violence.”^7^ It was said that “she had no say over her own body.” One of their children described him as “a monster.”^8^ She had six more children with him after her conviction, commenting that as a mother to see your babies hurt is “a private hell.” She and Brownie had “a lot of rumbles because I was just running and trying to stop him from hitting the kids.”^9^

Every time Terri managed to leave Brownie, he would violently reinstate himself in her life by coming after her and the children in the company of male associates, with guns, and coerce her into “returning”. She ran “ten times,” but he would find them, “and he had an army with him.”^10^

Brownie's coercive control was intended to undermine her sanity or possess her totally (although he was not successful in this). Terri described living a life of “fear, uncertainty, and chaos” where she doubted herself and thought, “God must hate me.” She reached the point that she was so “freaked out” that she thought Brownie could read her thoughts: “cause he even used to tell me that ‘I even know your thoughts’”.^11^

The psychologist's report, submitted to the Minister of Justice, described how Terri attempted to keep herself and her children safe from Brownie's abuse—she became vigilant to Brownie's behavior cues, attempting to minimize triggers for conflict or criticism, responding to Brownie in a “passive” manner and seeking to appease him (for example, submitting to sexual activity before being forced). Terri even had to suppress her grief at Chantelle's funeral after thinking that Brownie had shushed her.

Understanding IPV as coercive control moves the focus from Terri's mental health issues to the coercive context created by Brownie. The issue becomes less about her deficiencies and more about his behavior. It also allows us to access a larger and more accurate picture of the abuse strategies Brownie employed, to see these as ongoing and to understand how they operated over time to foreclose Terri's exercise of autonomy. This means we can better understand the threat Brownie posed when she confessed to his crime to avoid her surviving child from being sent home alone with him. It also makes it clear that Terri was not safe from Brownie just because, at that moment, she was in a room with police officers and physically separated from him. The abuse she was experiencing was not over—it was ongoing and would continue in the absence of adequate system intervention to stop his violence.

## Entrapment: Including the Broader Social Context

Understanding IPV as entrapment (FVDRC, 2016; Ptacek, 1999, p. 10; *R v. Ruddelle*, 2020; Tolmie et al., 2018; Wilson et al., 2019) means that we cannot understand a victim-survivor's experience of IPV without careful investigation into the following:

1. The operation of intersectional inequities and state-sanctioned violence in the victim-survivor's and their relational support network's lives (within an infrastructure of colonial violence in settler states). This includes understanding whether and how these exacerbate their abusive partner's ability to coercively control them and impact the safety responses available to them.
2. The realities of the family violence safety responses available to the victim-survivor—this includes the responses of the immediate community surrounding the victim-survivor and their abusive partner and of those agencies charged with assisting them. The question here is whether the victim-survivor and those who matter to them have access to equitable and dignifying systemic safety responses that are effective in addressing the coercive controlling behaviors of their abusive partner.
3. The abusive partner's coercive and controlling behavior and the impact of that behavior in closing down the victim-survivor's space for action (as discussed above).

An entrapment lens means that, in addition to understanding the abusive tactics of the person using violence, we are required to be properly cognizant of the *social context* in which the victim-survivor and their abusive partner are located. Bringing this broader context into focus ensures that we are squarely focused on the victim-survivor's circumstances rather than on their mental health issues. Like coercive control, IPV entrapment is experienced as an ongoing process and takes effect in a cumulative and compounding manner in people's lives.

Stark (2007) himself used a limited entrapment framework in explaining coercive control. He was clear that the tactics of coercive control used by men with their female partners utilize heterosexual gender norms and the gendered distribution of resources, and thus the broader social and systemic forces that support gender inequality (p. 21).

In acknowledging heterosexual sex/gender norms, however, it is generally assumed that these operate in a Western and non-intersectional manner. In fact, such norms are culturally specific and influenced by the shared history and circumstances of communities. For example, Wilson et al. (2021) point out that Māori gender norms cannot be flattened and distorted into Western notions of romantic love, sexuality, and the nuclear family. Wāhine Māori operate according to the central cultural values of *aroha* (compassion, empathy, and respect) and *manaakitanga* (sharing and caring for others). Wāhine draw on these values in the context of the lived reality of ongoing colonization and racism in Aotearoa New Zealand, as collectively experienced by Māori. Manaakitanga is therefore practiced with the knowledge that Māori women have of the damage also inflicted upon Māori men because of these violent legacies and their belief that these men have the potential to heal.

Women play a central role in holding together communities that are decimated by colonization and racism—absorbing the personal costs of performing this important work for the greater collective good. Significantly, women may be relied upon by state agencies to perform such a role even when those agencies are aware that this requires women to have contact with men who are extremely dangerous to them (see Douglas et al., 2020, pp. 499–501).

These examples highlight the first dimension of entrapment (as ordered above)—the necessity of understanding how structural inequities related to race, class, poverty, ableism, and heterosexism *intersect* with gender and exacerbate an abusive partner's ability to coercively control the victim-survivor. This occurs in multiple ways, depending on how these inequities have shaped service provision and approaches to addressing and healing from violence.

It is important to emphasize that an intersectional approach is about focusing on the “-isms” (racism, classism, sexism, ableism, heterosexism etc) as *interlocking social structures* that perpetuate inequity, not as *individual identity markers* (Sokoloff & Dupont, 2005) that are additive (Richie et al., 2021, p. 252). These interlocking structural inequities reproduce social relations of violence, and exclusion which oppress people collectively, not simply as individuals. Collins (2000) suggests that intersectional oppressions form a matrix of domination—a point that is critical because it reveals “the overall social organization within which intersecting oppressions originate, develop, and are contained” (pp. 228–229). In other words, attention needs to be focused on the *scaffolding* that encases the multiple axes of oppression. In settler states, colonization is not simply one of these axes; rather, it is an infrastructure that shapes the matrix itself.

As noted earlier, the second dimension of entrapment (as ordered above) requires a realistic understanding of the quality of the formal and informal safety responses available from those around the victim-survivor and their abusive partner (including the inequities that shape those responses). This is an essential component of the experience of IPV for victim-survivors because it is not simply the abuse strategies of their partner or the victim-survivor's decisions that will determine whether they and their children have access to safety. If the responses of those around the victim-survivor and their partner support his violence, are inadequate to address his violence, deplete her resources and/or cause harm to her or those who are important to her, then these responses make her less safe. Victim-survivors’ tactical responses to their circumstances are shaped by their partner's, as well as their community's and the system's patterns of harm.

Using an entrapment framing ensures that this social context is not dropped from our analysis. When we do not use an entrapment framework, then we risk erasing these broader operations of power and harm. It then becomes easy to see the problem as a problem of individuals - one individual man (or person using violence) who is behaving badly, but also the individual victim-survivor and their poor choices, substance abuse issues, and mental illness. Failing to realistically examine whether the safety responses available to the victim-survivor can address the harm their partner poses supports the erroneous assumption that these responses are excellent, and if only they had made the right “choices,” then they would be safe (Aikin & Goldwasser, 2010). This causes us to lose sight of the fact that if the available safety responses are not capable of addressing the pattern of harm that a victim-survivor experiences, then those responses are part of the ongoing process of entrapment in the abuse. Failing to examine the adequacy of the safety responses available from others supports what various scholars have theorized as victim-survivors being “responsibilized” for their abuse, essentially placing the onus on them and not the family violence safety system and/or their community for managing their partner's dangerousness (Barlow & Walklate, 2022; Grant, 2015).

It is probably fair to say that the state’s crisis response to family violence for those victim-survivors who need to use it does not match the operation and harm of IPV in any jurisdiction at this point. At the most basic level, the traditional Western toolkit for responding to IPV when a victim-survivor seeks formal support is obtaining a protection order, providing them with temporary refuge accommodation or charging the offender with a criminal offense. These are victim-survivor-initiated, one-off interventions that do little or nothing to manage patterns of ongoing harmful behavior. For example, a protection order is a court order *which must be breached before it triggers a safety response*, if the breach does trigger a safety response. A criminal prosecution is *a reaction to a past event*, which must be proven to have occurred to a high standard to result in a conviction. Sentencing the offender for proven past behavior is not the same thing as providing a safety response for the victim-survivor—victim safety is generally neither a mandatory nor a priority consideration in that process. Accommodation in a refuge may provide a victim-survivor a brief but life-disrupting respite, but it is not a long-term safety solution. This is not to discount the fact that there are jurisdictional attempts to develop holistic wraparound responses to family violence that go beyond these traditional approaches (Hester et al., 2019; Wehipeihana, 2019).

Because IPV comprises an ongoing pattern of retaliatory harm, unhelpful responses are potentially dangerous because they alert the person using violence to the victim-survivor's help-seeking, without providing them with effective protection from retaliatory violence. A criminal justice response, for example, that involves interviewing an abusive partner about the victim-survivor's allegations and then releasing him back into the community in circumstances in which he can readily access her if he is sufficiently determined to do so, is not a safety response.

Aside from the immediate state crisis response to family violence, victim-survivors may experience issues in using the legal system (family law, child protection, criminal law, and the migration and visa system) when trying to leave their abusive (ex)partners and/or address the abuse. Douglas (2021) coined the term “legal systems abuse” to describe how victim-survivors say their abusive (ex)partners “hunted, battled, and played with them through law” (p. 102).

Beyond these generalities, the level of social response for any victim-survivor will depend on their positionality at the intersection of different oppressions and the realities of their life circumstances. For example, indigenous women and women subjected to intersecting oppressions are likely to be dealing with multiple “systems of harm” (Larasi & Jones, 2017, p. 6), which means because of systemic inequities, they are less likely to have access to culturally safe services and are more likely to be pathologized and to experience extremely unsafe police and child protection service responses.

In Aotearoa New Zealand, more than half (54.7%) of the women living in New Zealand reported experiencing lifetime IPV exposure. However, for Māori women, it is almost two in every three (64.1%) (Mellar et al., 2023). The prevalence rate for Māori women is likely to be underreported in official data because of the underrepresentation of younger women in the New Zealand Family Violence Survey (NZFVS) and Māori being a young population (with a mean age of 27.6 years compared to 37.4 years for the NZ population). Māori women bear a greater burden in terms of the severity and lethality of violence. They are twice as likely to experience severe physical violence (30.9%) compared to New Zealand European women (14.4%) and experience notably higher rates of sexual violence (20.7% vs. 12.8%), controlling IPV (34.7% vs. 20.3%), and economic abuse (22.6% vs. 15.6%), although they have a similar prevalence rate for lifetime psychological IPV (54.8% vs. 51.6%) (Mellar et al., 2023). A similar overrepresentation exists in homicide cases, with Māori women being three times more likely to be victims of homicide. The Family Violence Death Review Committee (2017) reported that 98% of women in IPV-related death events were primary victims of their male partners in the preceding relationship. Notably, Māori comprise 50% of family violence-related homicides while only making up 16.5% of the population.

In Aotearoa New Zealand, wāhine Māori experiencing IPV describe responses from the agencies charged with assisting them that were more harmful than their partner's abuse (Wilson et al., 2019). For example, removing their children into state care with extremely damaging consequences for whānau Māori. These wāhine said they did not feel vulnerable until they were obliged to deal with state agencies: “Wāhine reported the focus of agencies as primarily deficit-focused and reflecting a victim-blaming approach” (Wilson et al., 2019, p. 32). Some Indigenous scholars have used the term “systemic entrapment” in order to highlight the unique nature and quality of the social responses to victim-survivors who belong to communities dealing with sustained and intergenerational experiences of state violence and harm (Wilson et al., 2019). These victim-survivors are not simply dealing with institutional indifference and failure at the point of crises but with broader, long-term community patterns of “containment and condemnation” across a range of different government systems and carceral institutions (Longbottom, 2020).

Migrant women seeking support for family violence have also been documented as facing geographic isolation from support systems, language barriers, as well as having insecure legal status, being financially dependent on their abusive partners, and lacking knowledge as to how the family violence safety system operates and what support is available (Graca, 2021). Graca (2017) noted that these women frequently navigate “varied and complex social and cultural conditions” and, due to their experiences in their countries of origin, can fear police and state interference “to such an extent” that they adopt “inconspicuous and informal approaches to protect themselves from abuse” (Barlow & Walklate, 2022, pp. 50–51).

Bumiller (2008) described how the state violence and neglect some victims-survivors experience replicates the IPV they are attempting to escape. These “‘victims’ enter into a perilous involvement with the penal/welfare state” and “often find that they experience brutalities that mimic the violence they hoped to leave behind” (p. 97).

Entrapment also requires consideration of the responses of those in the victim-survivor's and their abusive partner's immediate communities. For example, the abusive partner's pattern of harmful behavior does not exist in isolation from his wider position of power in society. It is relevant to know that he is close friends with the local police officer or that he has associates spread throughout her community network who will enforce his authority over her even when he is incarcerated. Victim-survivors may be in relationships with men who, because of their associations with other men, are extremely powerful and well-resourced. These women are facing coercion not just from one abusive man but from patriarchal collectives of men (Havard et al., 2023) that may even permeate the systems of law enforcement.

Entrapment is not a radical or new concept. Critical race and Indigenous feminist scholars, community activists, grassroots organizations, survivors, and reform bodies have for decades been documenting how entrapment operates in relation to family violence (Balzer et al., 1997; Ptacek, 1999, pp. 7–15; Richie, 1996). Yet these broader experiences of entrapment do not seem to form part of our understanding of *what IPV is*. This means that when it comes to responding to individual cases or developing policy responses to IPV, this huge body of knowledge falls away and often does not register in “our” individual and collective reactions. These acts of disappearing speak to who occupies positions of power in government, NGOs, and the academy, and the historical privilege that enables such “forgetting”.

While understanding IPV as coercive control represents an improvement on traditional responses, it still limits what we can see to the abuse strategies of individuals using violence and some gendered effects of white middle-class heterosexual gender norms. It fails to render visible the larger social context, other than as special “vulnerabilities” belonging to individual victim-survivors or groups of victim-survivors. Two examples illustrate this point: First, the legal response to victim-survivors of family violence who offend in response to their victimization, and second, the criminalization of coercive control.

In relation to the first issue, victim-survivors of IPV can be prosecuted for failing to protect their children from their partner's use of violence, participating in his criminal offending, offending under his coercion, and using violence themselves in self-protection or resistance. Victim-survivors do not offend, however, simply in response to individual incidents of abuse. In other words, they are not responding simply to the physical violence they are experiencing from their partner in the moment in which they offend. In making decisions to act, victim-survivors interpret what he is doing at the time in the light of what he has done before, what she knows he is capable of doing to her and others, and what level of social and system responsiveness she can count on.

However, when victim-survivors come up for trial, traditional criminal justice responses have assessed their criminal culpability as though they are only being abused in the moment when they are under direct physical attack from their male partner and have assumed that, in between these assaults, they have access to effective safety options (Tolmie et al., 2018). If the concept of coercive control is used correctly at trial, then legal decision-makers would be judging victim-survivors with a realistic understanding of the nature of the threat they face from their abusive partner and the limitations he has managed to place on their autonomy, including their capacity to seek help. However, this still leaves out significant information that decision makers need to accurately assess whether the victim-survivors’ actions were reasonable in their circumstances for the purpose of raising one of the criminal defenses. In the context of self-defense, for example, in the absence of an entrapment framing, defense lawyers and judges may not even notice that the Crown has failed to discharge its burden of proof on the facts. This occurs when the Crown asserts in court, *without providing any evidentiary basis for their assertion*, that leaving the relationship or calling the police would have provided the victim with safety, meaning that her use of violent self-help is unreasonable (Tolmie, 2020, pp. 19–20). If an entrapment framing is used, an investigation into whether there were, in fact, realistic safety options in the victim-survivor's actual circumstances is required, and any assertion to this effect by the prosecution requires supporting evidence. The absence of an entrapment framing may also make it more difficult for the court to understand that victim-survivors resist abuse—that their actions, including compliance, are always the result of strategic decisions evaluating the balance of risk for themselves and their children.

It is, of course, those victim-survivors who experience the most serious levels of entrapment, including systemic racism and structural violence, who are most likely to find themselves facing criminal charges because they are less likely to have access to safe and legal alternatives to using physical force to defend themselves or to acting under coercion (see, for example, FVDRC, 2017, p. 54). It follows that if we embrace coercive control without placing it in an entrapment framework, then we have not done enough to register these women's voices as reasonable in the criminal law context.

In relation to the second issue, the clearest policy response to the recognition of IPV as coercive control in a number of jurisdictions has been the creation of a new offense criminalizing coercive control. Criminalization as a strategy for responding to IPV is problematic for all victim-survivors because the criminal law serves the “interests of the state rather than the interests of either individual women or women as a group” (Barlow & Walklate, 2022, p. 64), and as noted above, a criminal justice response is not the same thing as a family violence safety response.

However, criminalization is particularly problematic for victim-survivors who belong to communities that have already been grossly over-criminalized. Women from these communities may want less rather than more state control in the lives of themselves and their children, are prone to being prosecuted themselves, and not infrequently may experience the criminal justice response as compounding their entrapment in the abuse. Furthermore, a criminal justice response to their abusive (ex)partner, who may also be the father of their children, may not be the type of justice response they are seeking. Victim-survivors from oppressed groups want their partners to stop using violence, but this does not mean they want to potentially risk their or their partner's lives and well-being by involving a racist and punitive criminal justice system. The hyperincarceration and brutalization of ethnic minority men has a devastating effect “on the mental well-being of individuals and on the social structure of communities,” devastation that, in turn, creates further risk for IPV (Coker & Macquoid, p. 587).

The creation of an offense of coercive control constructs IPV as an issue of bad individuals who must be punished (Aikin & Goldwasser, 2010; Coker & Macquoid, 2015), as opposed to the gross underinvestment by the state for many years into the safety of women and children,^12^ the social systems that we have in place that grant impunity to male violence in certain contexts (Richardson, 2016), and the underlying structural forms of violence that place women in precarious social positions and render them at risk of IPV. Institutionally racist carceral responses, as well as being directly harmful, divert resources from other possibilities, such as more effective safety responses (Family Violence Death Review Committee, 2016, pp. 64–85) or alternative transformative justice responses to address and heal from violence (see Kim, 2018; in Aotearoa New Zealand, for example, there are calls to revert to an indigenous justice system, Tolmie et al., 2022, p. 79). Richardson (2016) has said that indigenous women want healing for their men and themselves, as the “brutal history of colonial/state violence” targeted both men and women (p. 260).

Focusing on coercive control, rather than the broader processes of systemic entrapment, obscures the fact that a strategy of criminalization relies on the very institutions and practices that are part of *systems of harm* for particular victim-survivors, their abusive partners, and communities.

### Case Example: Terri Friesen

In Terri's case, the police were put on notice that they were dealing with a family violence homicide. An appropriate safety response by the police on this set of facts required cognizance that there could well be other victims. In this case, there would be a strong likelihood this was the mother (given typical gendered patterns of harm) and/or the sibling of the deceased child.

However, instead of a careful investigation, including attempts to gain the trust of any surviving victims to wrap support around them, the police put the *adult victim-survivor* under pressure to confess to the lethal abuse of her partner by *threatening* her surviving child (who had also been abused). In other words, instead of providing an adequate safety response to this whānau, they bolstered the coercion of the person using violence with their collective coercion as representatives of the state. A better safety response at this point may have avoided not only Terri's false confession but also the decades of additional abuse to which she and her tamariki were subjected. Understanding this reveals significant responsibility on *the Police* themselves, as an arm of the state, for coercing Terri's false confession.

At a structural level, Terri was faced with an institutionally racist criminal justice system (Tauri, 2019) and child protection system, which even then was known to be unsafe for tamariki Māori and their whānau (Māori Perspective Advisory Committee, 1988). As the work of the Royal Commission of Inquiry into Abuse in Care (2021) has evidenced, tamariki Māori were more likely to be removed from their whānau into state care. When in the care of the state, they were at high risk of being physically and sexually abused by adults, with no substantive action taken by the state to prevent or stop this abuse, despite the abuse being common knowledge (Savage et al., 2021).

Terri wanted to safeguard Rebecca from further abuse, either potentially by the state or by Brownie. In the absence of the police acting protectively toward Rebecca or Terri, Terri had to navigate a safer place for her daughter. Her space for action was extremely constricted. The most obvious way she could protect Rebecca was to falsely confess to manslaughter. This confession had nothing to do with mental health or experiences of trauma compromising her decision-making, and everything to do with a mother wanting to safeguard her remaining daughter while entrapped in a system of ongoing harm.

The unsafe responses Terri received from those entrusted to protect her were not limited to this event. They include the judgments of the medical staff after Chantelle was born, who failed to account for the poverty and difficult life circumstances Terri was struggling with and so saw her as a neglectful mother rather than a mother facing impossible circumstances.^13^ They also included the decision of the police not to reopen the case in 1991 (which gave Brownie a sense of invincibility and compounded her entrapment) and the failure to automatically overturn Terri's conviction when it was acknowledged in 2002 that she had wrongly taken the blame for Brownie's violence. Most shockingly, Terri described how the community and agencies were complicit in Brownie's abuse of her and her children. The first time she escaped Brownie and entered a refuge with her four tamariki, she had just arrived when the phone started ringing. The refuge worker said her that it was for her. It was Brownie on the line, saying “And he's like ‘you bitch, I know where you are, got a double barrel I’m coming for you.’”^14^ Terri described being too afraid to leave the building and relying on other women to shop for her. One night, the staff and other residents left, and Terri was alone all night with her kids, jumping at every sound. She also described the “army” with weapons that accompanied Brownie to get her every time she managed to flee.

## Conclusion

In this article, we suggest that a failure to locate coercive control within a broader entrapment framework means using a lens to understand IPV that does not capture the full picture of that violence for victim-survivors. The most impacted victim-survivors are those who have the largest chunks of their experience left out of our understanding of their situation—these are women for whom the experience of IPV entrapment is most extreme. These are also the women who most need equitable safety and justice responses to family violence. It follows that our responses to IPV will not work for women and, unacceptably, they will be actively deleterious for some.

Understanding IPV as coercive control, without locating this understanding within an entrapment framework that accounts for more than gender, is an approach that seeks first and foremost to secure (white) women's safety from men's violence (Moon & Holling, 2020); achieving this through the construction of carceral states, while remaining silent on how colonial and structural forms of violence continue to transgress the safety of indigenous women and their communities, as well as other groups of people targeted for oppression. White feminism needs to be accountable for the continued epistemic violence involved in such an approach (Moreton-Robinson, 2021).

Decolonization requires a “trickle-up” (Nichols, 2013) not a trickle-down approach to justice. Million (2009) points out that “‘decolonize’ means to understand as fully as possible the forms colonialism takes in our own times” (p. 55). This does not mean continuing to discuss “minority” women's and communities’ experiences of structural violence in the margins, but rather getting on with the work of constructing conceptualizations of IPV that are not narrow, inaccurate, and ultimately harmful. We are therefore suggesting that policy makers, those embarking on legislative reform and practitioners from all disciplines understand and respond to IPV as a form of social and systemic entrapment.

## Notes

1. Kelly Phillips, “How Terri Friesen, convicted of killing her baby, finally cleared her name” 11 September 2018 The Spinoff.

2. Kelly Phillips, “How Terri Friesen, convicted of killing her baby, finally cleared her name” 11 September 2018 The Spinoff.

3. Letter from Phil Hamlin (17 November 2020) to the Minister of Justice at p. 2.

4. Letter from Phil Hamlin (17 November 2020) to the Minister of Justice at p. 3.

5. Letter from the Minister of Justice, Hon Kris Faafoi (8 April 2021), at p.2.

6. Letter from Tim McKinnel (13 August 2020), at p. 2.

7. Letter from Tim McKinnel (13 August 2020), at p. 2.

8. *I am Innocent*, Screentime New Zealand, 2015.

9. Testimony from Terri, *I am Innocent*, Screentime New Zealand, 2015.

10. Testimony from Terri, *I am Innocent*, Screentime New Zealand, 2015.

11. Testimony from Terri, *I am Innocent*, Screentime New Zealand, 2015.

12. Kate FitzGibbons has highlighted the billions that have been spent on terrorism, with comparatively little spent on addressing domestic terrorism in the form of family violence: Conference paper, ANZSOC 31st Conference, *Encountering Crime: Doing Justice*, University of Melbourne, 5–7 December 2018.

13. For example, when Terri was tried for killing her child the medical staff at the hospital shared their judgments about Terri's failures to regularly visit Chantelle when she was at the hospital in the 4 weeks after the birth. In fact, she was reliant on public transport, sometimes not having the fare, sometimes not being able to make one of the two buses that ran to the hospital. She had a two-and-a-half-year-old that she was not able to leave in the care of her abusive stepfather and was trying to prepare her house for a newborn.

14. Testimony from Terri, *I am Innocent*, Screentime New Zealand, 2015.
